# Myelin Oligodendrocyte Glycoprotein Optic Neuritis Presenting With Orbital Apex Syndrome

**DOI:** 10.7759/cureus.38975

**Published:** 2023-05-13

**Authors:** Farhana Nabila Sulaiman, Nur Farhana Kamardin, Mohamed Iliyas Sultan Abdul Kader, Hannie Ch'ng, Wan Haslina Wan Abdul Halim

**Affiliations:** 1 Department of Ophthalmology, Faculty of Medicine, Universiti Kebangsaan Malaysia, Kuala Lumpur, MYS; 2 Department of Ophthalmology, Hospital Selayang, Selangor, MYS; 3 Department of Ophthalmology, Hospital Kuala Lumpur, Kuala Lumpur, MYS; 4 Department of Otorhinolaryngology-Head and Neck Surgery, Faculty of Medicine, Universiti Kebangsaan Malaysia, Kuala Lumpur, MYS; 5 Department of Otorhinolaryngology-Head & Neck Surgery, Hospital Melaka, Melaka, MYS

**Keywords:** myelin oligodendrocyte glycoprotein (mog), myelin oligodendrocyte glycoprotein antibody disease, anti-mog antibody, optic neuritis, orbital apex syndrome

## Abstract

A 36-year-old man presented with an acute onset of a right eye monocular altitudinal defect associated with pain on eye movement upon waking up from sleep. His right eye subsequently developed outward deviation and a total loss of vision. Clinical examination of the right eye revealed a visual acuity of no light perception (NLP) with the presence of relative afferent pupillary defect (RAPD) and involvement of cranial nerves II, III, IV, and VI. A marked optic disc swelling and peripapillary hemorrhages were seen in the right fundus. Contrast-enhanced computed tomography of the brain and orbit showed a unilateral enlargement and enhancement of the right intraorbital and intracanalicular segments of the optic nerve with surrounding fat stranding and orbital apex crowding. Magnetic resonance imaging showed T2/fluid-attenuated inversion recovery hyperintensity and enhancement of the optic nerve and the myelin sheath. Serum anti-myelin oligodendrocyte glycoprotein antibodies were detected. He was treated with corticosteroids, plasma exchange, and intravenous immunoglobulin. His vision improved slowly after treatment. This case report shows the diverse manifestations of myelin oligodendrocyte glycoprotein antibody disease, which includes the orbital apex syndrome.

## Introduction

The hallmarks of orbital apex syndrome are vision loss from optic neuropathy and ophthalmoplegia with multiple cranial nerve involvement. Orbital apex syndromes may occur due to infection, inflammation, malignancy, vascular, trauma, and iatrogenic causes [1]. Here, we report the case of a 36-year-old man with visual field defect, visual loss, and ophthalmoplegia associated with positive anti-myelin oligodendrocyte glycoprotein (MOG) antibodies which showed visual improvement after commencing treatment.

## Case presentation

A 36-year-old man with no comorbidity presented to the eye clinic with a right eye sudden-onset, monocular altitudinal defect with pain on eye movement upon waking up from sleep. It was associated with exotropia of the right eye. One day after the presentation, he developed total visual loss in the right eye. This was the first episode of symptoms. He had no diplopia or symptoms of raised intracranial pressure. There was no significant past ocular history or family history of ocular diseases.

**Figure 1:**
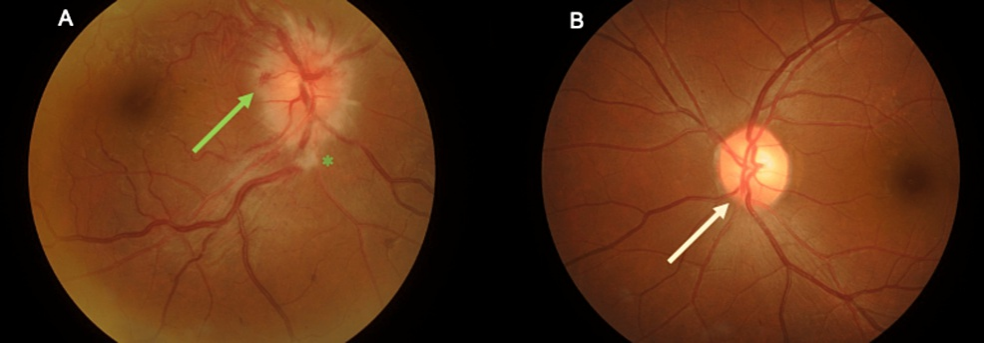
Bilateral fundus photo of the right eye (A) and the left eye (B). A: The optic disc of the right eye was swollen with flame-shaped hemorrhages and dot hemorrhages adjacent to the optic disc (green arrow). There was a cotton wool spot (green asterisk) inferior to the optic disc. B: The left optic disc (yellow arrow) and fundus were normal.

**Figure 2:**
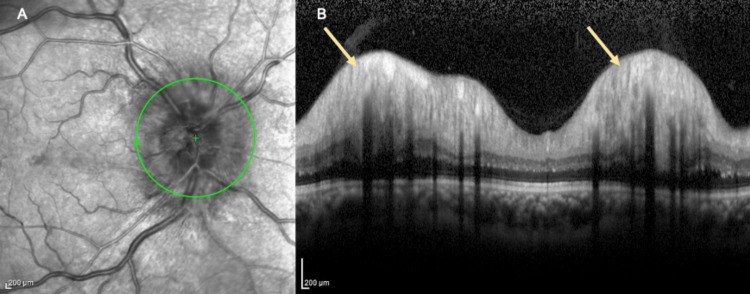
Ocular coherence tomography of the right optic disc. A: Ocular coherence tomography showed marked swelling of the right optic disc (green circle). B: Ocular coherence tomography showed marked papillary elevation of the right optic disc (yellow arrow).

Ocular examination revealed a visual acuity of no light perception (NLP), with a positive relative afferent pupillary defect (RAPD) in the right eye. There was a right eye exotropia of 15 degrees and extraocular movement limitations in all gazes predominantly in adduction. There was no ptosis or proptosis. The optic disc of the right eye was swollen with flame-shaped hemorrhages and dot hemorrhages adjacent to the optic disc (Figure 1A). There was a cotton wool spot inferior to the optic disc, and Paton’s line was present temporal to the optic disc. The macula was normal. There was no vasculitis, retinitis, or vitritis. The left eye visual acuity was 6/9, and pinhole was 6/6. The left optic disc and fundus were normal (Figure 1B). Anterior segment and intraocular pressures were normal bilaterally. There was no anisocoria, and no ocular or carotid bruit was heard. Cranial nerve examination revealed multiple impairments in cranial nerves II, III, IV, and VI. Other neurological and systemic examinations were unremarkable. Ocular coherence tomography (OCT) showed marked swelling and papillary elevation of the right optic disc (Figures 2A, 2B). OCT of the left optic disc showed a normal contour. Bjerrum visual field test was attempted but unsuccessful. Visual evoked potential testing showed prolonged P100 latencies over the right eye. There was electrophysiological evidence of a defect in the anterior visual pathway of the right eye.

Serology analysis via cell-based assay revealed positive serum anti-MOG antibodies. The coagulation profile, erythrocyte sedimentation rate, and C-reactive protein were normal. Other laboratory investigations such as serum anti-aquaporin-4 (AQP4) antibody, complement C3, complement C4, anti-neutrophil cytoplasmic antibody (ANCA), anti-nuclear antibody (ANA), and urinalysis were normal. Infective screening such as venereal disease research laboratory test, hepatitis B virus surface antigen, hepatitis C virus antibody, and human immunodeficiency virus antibody was non-reactive. Thrombophilia screening showed normal results. Other hematological and biochemical laboratory investigations were normal. Mantoux test and chest X-ray revealed normal findings.

**Figure 3:**
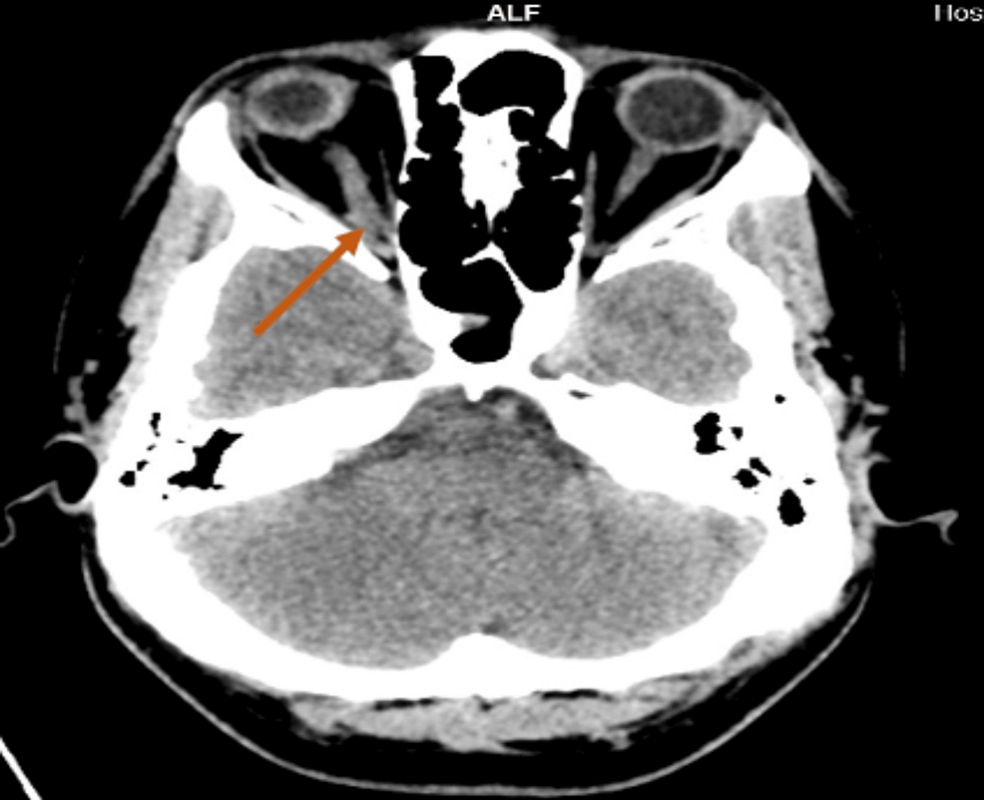
Contrast-enhanced computed tomography of the brain and orbit (axial view). Arrow: Contrast-enhanced computed tomography of the brain and orbit revealed a right unilateral enlargement of the right intraorbital and intracanalicular segments of the optic nerve, with orbital apex crowding compared to the left optic nerve. The right optic nerve demonstrated heterogeneous enhancement with surrounding fat stranding.

**Figure 4:**
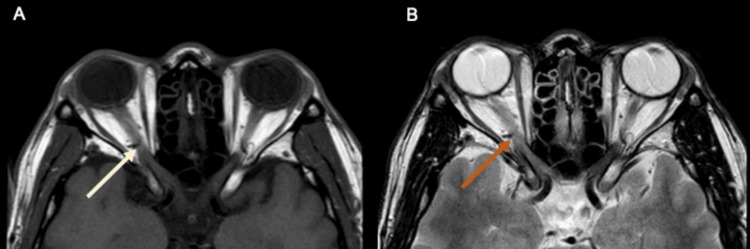
Magnetic resonance imaging of the brain/orbit at T1 (A) and magnetic resonance imaging of the brain/orbit at T2 (B). A: Magnetic resonance imaging of the brain/orbit at T1 showed thickening of the right optic nerve (yellow arrow). B: A thickened right optic nerve with a hyperintensity seen on T2/fluid-attenuated inversion recovery with mild T2 hyperintensity of the myelin sheath (red arrow).

Contrast-enhanced computed tomography of the brain and orbit revealed a unilateral enlargement of the right intraorbital and intracanalicular segments of the optic nerve measuring 0.5 cm (0.3 cm on the left), with orbital apex crowding compared to the left optic nerve. The right optic nerve demonstrated heterogenous enhancement with surrounding fat stranding (Figure 3). The right cavernous sinus showed mild effacement. The findings were suspicious of optic neuritis. Magnetic resonance imaging (MRI), magnetic resonance angiography, and magnetic resonance venography with intravenous gadolinium of the brain, orbit, and the whole spine showed thickening of the right optic nerve measuring 5.4 mm in diameter (left optic nerve measures 4.1 mm) with a hyperintensity seen on T2/fluid-attenuated inversion recovery with a mild enhancement of the myelin sheath (Figures 4A, 4B). The inflammatory changes of the right optic nerve were in keeping with right optic neuritis. Lumbar puncture results showed no atypical or malignant cells for cerebrospinal fluid (CSF) cytology. CSF was clear with zero cell count. The patient had negative CSF anti-MOG antibody, oligoclonal bands, and anti-AQP4 antibody. There was no growth of CSF cultures. CSF *Mycobacterium tuberculosis* polymerase chain reaction and Ziehl-Neelsen acid-fast bacilli smear also showed negative results.

The patient was subsequently administered intravenous methylprednisolone (IVMP) 1 g daily for five days. Upon completion of the IVMP, visual acuity slowly improved to hand movement. There were no more eye pain and extraocular movement restrictions. The patient was started on oral prednisolone 1 mg/kg for two weeks. One week after discharge, the patient developed bilateral central serous chorioretinopathy evidenced by a focal area of subretinal fluid on OCT due to complications of oral prednisolone. Oral prednisolone was tapered down slowly and the patient was given oral azathioprine 100 mg daily. Five cycles of plasma exchange (PLEX) and intravenous immunoglobulin (IVIG) therapy with a dose of 33 g daily over five days were initiated sequentially by the neurology team. After completion of PLEX and IVIG treatment, the right eye visual acuity improved to 6/60. There was no more visual field defect. However, color discrimination and light brightness were impaired.

Three months after the first presentation, the visual acuity of the right eye improved to 6/36 with persistent impairment in color discrimination and light brightness. Fundus examination revealed a pale optic disc with a cup-to-disc ratio of 0.4 over the right eye. Bjerrum test revealed no visual field defects. The ocular motility impairment was completely resolved. There was no optic neuritis relapse or other neurological issues. He was subsequently lost to follow-up after the final visit.

## Discussion

Myelin oligodendrocyte glycoprotein antibody disease (MOGAD) has a prevalence of 0.12 per 100,000 in the Malaysian population, with a male predilection of 2:1, and affects a younger age group of 23.8 ± 14.4 years [2]. The patient in our case report was 36 years old, which falls into the age group for MOGAD. MOG optic neuritis (MOG-ON) is immune-mediated and categorized under atypical optic neuritis, which is less likely to have a spontaneous resolution compared to multiple sclerosis-related optic neuritis. It is also necessary to differentiate optic neuritis from ischemic optic neuropathy caused by thrombophilia or vasculitic vascular occlusion [3]. In our case, due to poor presenting visual acuity, rapid progress, and deterioration, vascular causes were initially entertained and ruled out.

MOG is a central nervous system (CNS) membrane protein expressed on the oligodendrocytes, and damage to MOG causes anti-MOG syndromes [4]. It has been postulated that antibodies directed at MOG play a role in CNS demyelination [2]. Patients with MOG-ON typically present with pain on eye movement and optic disc swelling and may also be associated with peripapillary hemorrhages and wrinkles [5-7]. They may also present with frequent relapses and may require different treatment approaches [8]. Unilateral optic neuritis occurs in 49% of MOG-ON patients, with bilateral involvement being more common [6]. In this case, the patient presented with a first episode of unilateral visual field defect, pain on eye movement, and optic disc swelling with peripapillary hemorrhages on funduscopy.

The most common first clinical manifestation in MOGAD is optic neuritis. Although it has a severe presentation, after treatment, patients often have a good visual outcome [9]. Acute optic neuritis may present with any type of visual defect depending on the location of inflammation [10]. Relapsing optic neuritis with no CNS symptoms occurs in 30% of MOGAD patients, although they may develop seizures, myelitis, and encephalitis [5]. In the pediatric population, they typically manifest as acute disseminated encephalomyelitis [6]. MOGAD has also been associated with uveitis, pre-retinal macular hemorrhage, venous stasis retinopathy, neuroretinitis, acute macular retinopathy, nystagmus, diplopia, ocular flutter, papilledema, optic perineuritis, orbital apex syndrome, orbital inflammatory syndrome, and visual field defects [11]. The mechanism of MOGAD with cranial nerve involvement is hypothesized to be due to inflammation caused by MOG-secreted isoform triggering cranial nerves autoimmunity, pontine injury, or patient’s susceptibility to autoimmunity [12]. In patients with MOGAD for eight years or more, 93% have relapses [6].

On MRI, optic nerve enhancement is longitudinally extensive with perineural involvement in MOG-ON, usually not involving the optic tract and optic chiasm [5,6]. These findings were seen in our patient radiologically. In previous literature, there were several cases of MOGAD presenting with optic neuritis and orbital apex syndrome manifested with ocular motility abnormalities [13,14].

In our case, during the acute presentation of optic neuritis, the MOG antibody serology was positive. In acute episodes, the MOG antibody test is highly specific and sensitive for diagnosing MOGAD [15]. Prompt removal of systemic MOG antibodies is the principal aim of therapy in MOG-ON to achieve good outcomes [16]. The suggested IVMP dose in pediatric patients is 20-30 mg/kg/day; however, for adults, the suggested maximum dose of 1-2 g is given for three to five days [16]. A high dose of solumedrol of 1 g daily given intravenously for five days may also be used as steroid therapy. For patients with multiple relapses or suboptimal response to IVMP, IVIG is suggested with a dose of 2 g/kg over two to five days, or PLEX over five to seven days alternately [16]. Immunosuppressant therapy such as azathioprine, rituximab, and mycophenolate aids in minimizing the rate of relapse in MOGAD cases with relapse [5,6]. Long-term immune therapy will be required if a patient has more than one attack. Long-term follow-up is important for MOG-ON patients as the disease has a high incidence of relapses. However, our patient was lost to follow-up after the third month of the first presentation.

Only 6% of MOG-ON patients have a final visual acuity of 6/60 or worse as most patients have an excellent visual recovery with a mean visual acuity of 6/9 [5]. Axonal depletion or demyelination involving conduction block which begins in the acute stage determines whether there will be a permanent visual loss [3]. In this case, the late and delayed treatment of IVMP, PLEX, and IVIG in view of the atypical MOG-ON presentation of orbital apex syndrome may be the cause of the poor final visual outcome of 6/36.

## Conclusions

MOGAD may be associated with a diverse clinical spectrum including atypical MOG-ON presentations such as orbital apex syndrome and multiple cranial nerve palsy. IVMP is the first-line treatment for acute attacks. However, in patients with suboptimal response, IVIG and PLEX may be alternative options. The key to achieving good outcomes and retaining functional vision in MOGAD patients is prompt MOG antibody testing, urgent radioimaging for diagnosis, and initiating treatment as early as possible.
